# Analysis of multipath effects in global trade networks based on multimodal, high dimensional, heterogeneous transformer architecture: Deconstruction of nonlinear cascade and dynamic chain reaction

**DOI:** 10.1371/journal.pone.0328687

**Published:** 2025-08-06

**Authors:** Chengfu Fan, Shoujiu Xiong, Fei Zhou

**Affiliations:** School of Computer Engineering, Anhui Wenda University of Information Engineering, Hefei, Anhui, China; Hanyang University, KOREA, REPUBLIC OF

## Abstract

This paper proposes a multi-path effect analysis model for major economies in the global trade network based on the MH-DP Transformer architecture. The global trade network comprises interconnected nodes, with interactions among major economies (China, the US, Japan, and Europe) being crucial. A trade network model with four regional nodes and their connections is established to simulate trade flow propagation and capture the nonlinear feedback mechanism through an iterative process. The experimental results demonstrate that trade volume changes exhibit significant nonlinear trends, and mutual influences between economies produce strong cascading effects. For instance, China experiences significant fluctuations when interacting with the US and Europe, while Europe shows strong chain reactions with Japan. The model outperforms other comparative models, achieving the best path prediction accuracy (82.5%) and convergence speed, effectively capturing nonlinear effects. Furthermore, the paper uses 3D charts and heat maps to visualize global trade flow changes, offering a quantitative perspective on understanding the complexity of global trade interactions. The experimental results validate the model’s strong ability to analyze multi-path effects and dynamic chain reactions in global trade networks, providing a powerful tool for future economic policy formulation and interactive prediction in regional economic integration.

## 1. Introduction

With the advancement of globalization, the international trade network is becoming increasingly complex, and economic interactions between countries have formed complex multi-path effects. Understanding the nonlinear cascading effects and dynamic chain reactions in this network is of great significance for predicting trade shocks and optimizing global supply chains. In recent years, deep learning models based on Transformer architecture have demonstrated strong advantages in processing complex networks and large-scale temporal data [1]. These models can not only capture complex dependency relationships, but also reveal the interaction effects between nodes in the system [2]. For example, deep learning models have been widely applied in fields such as global trade flow forecasting and commodity flow analysis, providing powerful data support for policymakers and businesses [3]. Especially when dealing with large-scale data, the nonlinear modeling ability of deep learning models is particularly prominent, which can effectively handle multidimensional information including spatiotemporal data, economic factors, etc. [4]. In the global trade network, the mechanisms of multi-path effects and nonlinear cascading effects are key to predicting trade shocks and analyzing market risks. Through modeling global trade flows, energy markets, shipping networks, etc., researchers have found that these nonlinear effects are not just simple causal relationships, but complex interactions and feedback mechanisms [5]. These findings provide new ideas and methods for supply chain management and logistics optimization in global trade [6]. Therefore, exploring and understanding the underlying mechanisms of these effects is of great significance for enhancing the global economy’s responsiveness and flexibility.

This study aims to conduct a comprehensive analysis of multi-path effects in global trade networks by constructing a multimodal high-dimensional heterogeneous Transformer model. This model can capture the complex interactive relationships in global trade from different perspectives, thereby revealing the deep mechanisms of nonlinear cascading effects and dynamic chain reactions. In addition, with the advancement of data acquisition technology, fusion models based on graph neural networks (GNNs) and deep learning provide more accurate predictive capabilities, which have the potential to revolutionize the optimization of global trade networks [7].

## 2. Related work

### 2.1 Research progress on global trade networks

The research on global trade networks has gone through a transition from single country or regional trade patterns to global macro modeling. With the improvement of computing power, the research focus has gradually shifted from local trade relations to global trade network analysis. Early research mainly focused on the trade behavior and economic models of a single country or region, exploring how to understand and optimize trade flows through network topology [8]. As research deepens, scholars have begun to pay attention to the interdependence between nodes and their propagation effects in global trade [9]. These studies have revealed the interconnections and impacts of trade flows between different economies, particularly in the context of increasingly complex global supply chains and cross-border economic cooperation.

Despite significant progress in current research on global trade networks, there are still some areas that have not been fully studied, especially in modeling nonlinear effects and path interactions. The nonlinear characteristics of the global trade network and the complex path interactions that affect trade patterns have not received sufficient attention. For example, the flow of goods and services in global trade is often influenced by multiple factors, and traditional linear models are difficult to capture these complex relationships [10]. In addition, under the background of globalization, the formation and evolution of trade networks are driven by various economic, political, and technological factors, which have been largely overlooked in existing research. This may result in certain prediction biases in practical applications.

With the development of artificial intelligence technology, more and more research is applying advanced methods such as deep learning to optimize the analysis of global trade networks. For example, Wang, Jiang, and Shu (2022) proposed a carbon trading price prediction model based on improved deep learning methods, which is considered to have important application potential in the fields of energy and environmental trading in global trade [11]. Similarly, Su et al. (2024) used deep learning to predict and analyze the relationship between the futures market and the Baltic Dry Index, providing a new analytical tool for commodity price fluctuations in global trade [12]. In addition, using deep learning methods to study legal issues in international trade agreements is also becoming a new research direction [13].

However, despite the new ideas provided by artificial intelligence technology for the study of global trade networks, how to deal with the complexity and uncertainty in the global trade network remains a challenge. Most existing research focuses on how to improve the predictive ability and analytical accuracy of models, but there are still certain limitations in the interpretability of models and the handling of complex trade paths [14]. Future research needs to further explore how to introduce more dimensional factors such as cultural differences, political environment, and technological innovation into the complexity of global trade networks, in order to more comprehensively capture the evolutionary dynamics of global trade [15].

In summary, research on global trade networks is moving towards more complex and comprehensive directions, but still faces many challenges. Future research should further strengthen the in-depth exploration of nonlinear effects, path interactions, and the dynamic evolution of global trade networks, in order to better understand the complexity of the process of global economic integration.

### 2.2 Application of transformer architecture in complex network analysis

The Transformer architecture, due to its excellent self attention mechanism, has demonstrated significant advantages in processing large-scale temporal data and modeling complex networks. Through this mechanism, Transformer is able to capture the dependency relationships between different nodes on a global scale, making it particularly suitable for modeling dynamic evolution in complex networks. Compared to traditional Recurrent Neural Networks (RNNs), Transformer architecture is capable of parallel processing of information, resulting in higher efficiency and better performance in handling large-scale data [16]. In addition, the self attention mechanism of Transformer allows the model to focus more flexibly on different parts of the input sequence, further enhancing its performance in complex network analysis.

In recent years, the Transformer model has gained significant traction across various fields, particularly in network structure optimization, path propagation analysis, and node feature learning. These applications have demonstrated Transformer’s potential to address complex challenges in network analysis, but there are still areas that require further exploration, especially regarding multimodal heterogeneous data environments.For network structure optimization, Transformer’s self-attention mechanism has proven effective in uncovering intricate relationships between nodes in a network. Through its ability to capture dependencies across different parts of the network, it achieves more accurate optimization of network structures. This is evident in recent studies where Transformer models have been used to enhance network connectivity and robustness [17]. Unlike traditional methods, which may struggle to account for non-linear relationships, Transformer can dynamically adjust its attention weights, making it suitable for complex and evolving networks. However, while Transformer’s self-attention mechanism excels in these tasks, challenges remain in how it can be applied more efficiently when the network involves multiple data types and heterogeneous sources.In the realm of path propagation analysis, Transformer’s ability to model long-term dependencies is one of its key strengths. Traditional methods often face issues like information decay and the inability to capture dependencies over long distances within a network. Transformer addresses this by maintaining the flow of information across the entire network, preventing the loss of valuable contextual data. This capability allows Transformer to model more accurate and realistic path propagation, especially in dynamic networks where relationships between nodes evolve over time [18]. Nevertheless, despite its advantages, further research is needed to optimize Transformer’s handling of large-scale networks where path propagation is influenced by multiple layers of factors, such as changing external conditions or network disturbances.Node feature learning is another area where Transformer has shown remarkable potential. Through its attention mechanism, Transformer can discern subtle interactions between nodes, improving the learning of node features. This has been particularly useful in cases where the network’s nodes exhibit complex or sparse feature sets, as the model can adaptively focus on the most informative aspects of the data [19]. However, when it comes to multimodal heterogeneous data environments—where different modalities (e.g., images, text, and numerical data) are involved—integrating such diverse types of data to improve node feature learning remains a critical challenge. This is a gap that many existing models fail to bridge adequately, as they often struggle with issues of cross-modal alignment and data fusion [20]. The integration of multimodal heterogeneous data is a crucial aspect of the future of Transformer models in global trade network analysis. These environments often involve data with high levels of heterogeneity, making it difficult for models to learn from all available sources effectively. Research in this area is increasingly focused on developing techniques that improve the integration of diverse data types, reduce the information gap between them, and enhance the overall performance of Transformer models in practical applications. Recent studies have suggested that deep learning methods, such as attention mechanisms or cross-modal learning frameworks, can be leveraged to improve data processing flows, thus enabling better performance across diverse datasets [21–23]. However, the challenge remains in ensuring that these improvements lead to better generalization across different tasks, especially when faced with unseen or rare data types.Another critical area of development is the scalability of Transformer models to handle large-scale network data. While Transformer models have demonstrated strong performance in smaller or mid-sized networks, as the scale of the data increases—especially in the context of global trade networks—the computational complexity and memory requirements become significant obstacles [24–28]. The development of more efficient Transformer architectures, capable of handling massive amounts of data without compromising on performance, has therefore become a focal point of research. Innovations such as sparse attention mechanisms and hierarchical attention structures are some approaches being explored to address these issues and allow Transformer models to scale effectively. As the field advances, designing Transformer models that can manage large, heterogeneous datasets without sacrificing accuracy will be critical to improving their applicability in real-world, large-scale network analysis [29–33]. In conclusion, while Transformer models have demonstrated strong capabilities in network structure optimization, path propagation analysis, and node feature learning, several challenges remain in their application to multimodal heterogeneous data environments. Key areas that require further research include improving data integration, enhancing generalization ability, and designing scalable model architectures for large-scale networks. Addressing these challenges will be crucial for advancing Transformer-based models and expanding their practical applications, particularly in the context of complex global trade networks [34–38].

Overall, the application of Transformer architecture in complex network analysis still has broad research prospects and potential, but how to further improve its performance and ability to handle heterogeneous data in practical applications is still an urgent challenge that the academic community needs to overcome.

### 2.3 Research content and innovation of this article

**Fig 1:**
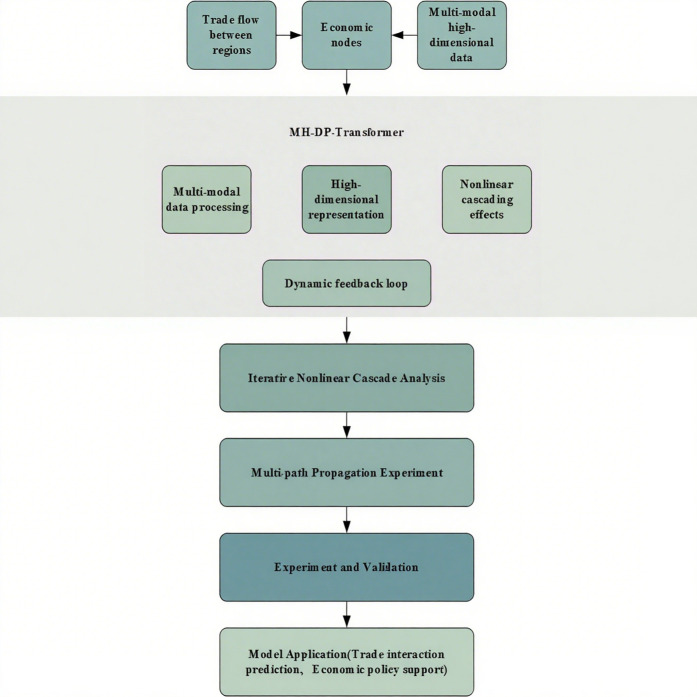
Technical roadmap of this article.

The research objective of this article mainly focuses on solving the complexity problems in the global trade network, aiming to reveal the nonlinear interactions and dynamic changes between global trade paths. To achieve this goal, this article will construct a comprehensive model based on multimodal high-dimensional data and Transformer architecture. The core issue of the research is how to better understand the multi-level interactions between countries in the global trade network and effectively extract and integrate information from heterogeneous data environments as Fig 1.To address these issues, this article will start with modeling nonlinear cascading effects and analyze how dynamic chain reactions affect the propagation of global trade pathways. By delving into the mechanism of path propagation, the research aims to propose a novel algorithm framework to enhance the performance of traditional network analysis methods in handling complex data, thereby better revealing the complex dynamic changes of global trade networks.

The innovation of this article is mainly reflected in the following three aspects:

(1) High dimensional feature extraction method for multimodal data fusion: In global trade network analysis, different data sources (such as import and export data, trade policies, macroeconomic indicators, etc.) have high heterogeneity. We propose an innovative multimodal data fusion method that effectively integrates various heterogeneous data and extracts high-dimensional features, thereby improving the prediction accuracy and generalization ability of the model. This method not only improves the efficiency of information utilization, but also provides a new perspective for studying the dissemination mechanism of global trade pathways.
(2) Optimizing Transformer architecture to adapt to complex network data: Although traditional Transformer architecture performs well in fields such as natural language processing and image recognition, it still has certain limitations when dealing with complex dynamic systems such as global trade networks. This article proposes a new model design by optimizing the Transformer architecture, enhancing its adaptability in multimodal data environments. By adjusting the self attention mechanism and enhancing the model’s ability to capture complex dependencies between nodes, we have effectively improved the performance of Transformer in complex network analysis.
(3) Dynamic modeling of nonlinear cascading effects: Path propagation in global trade networks is often accompanied by nonlinear cascading effects, which lead to complex changes in trade flows. We innovatively introduced an analytical method of dynamic chain reaction to model the mutual influence between global trade paths in detail. This method can capture the nonlinear interactions between trade paths, revealing how the global trade network spreads and adjusts through cascading effects under specific external shocks, providing more accurate decision-making basis for policy makers.

## 3. Model and algorithm design

### 3.1 Architecture design of multimodal high-dimensional heterogeneous transformer model

**Fig 2:**
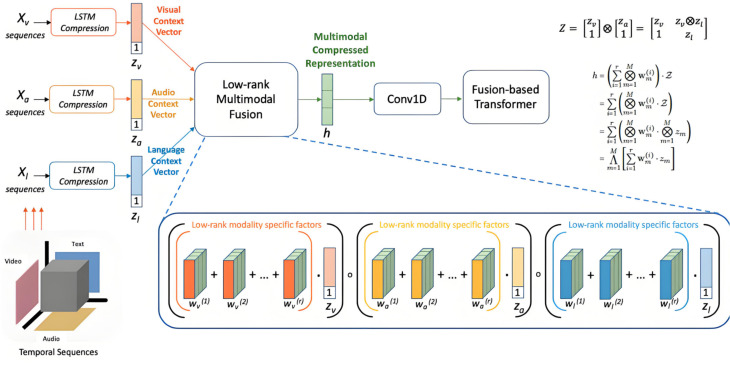
Multimodal transformer architecture.

The multimodal high-dimensional heterogeneous Transformer model proposed in this study aims to solve the analytical problems of multi-path effects and nonlinear cascading reactions in global trade networks. This model integrates multiple types of input data (such as national trade volume, industrial structure, geographical factors, historical transaction data, etc.), based on multimodal data, and uses fusion methods for data preprocessing to ensure effective interaction and synergy between different data types in the model, as shown in Fig 2.

The use of multimodal data fusion plays a critical role in this model. Global trade networks involve various types of data that differ in their nature (e.g., quantitative trade volume, categorical industrial classification, spatial geographical data), each offering different perspectives on the relationships within the network. To ensure these diverse data types interact efficiently, a fusion method is employed during the data preprocessing stage. This fusion ensures that the features from different modalities can be integrated meaningfully before they enter the Transformer model, preventing information loss and enabling cross-modal synergy. By aligning the different data sources effectively, the model becomes more robust, capable of learning comprehensive representations that consider all aspects of the global trade network simultaneously. This preprocessing step enhances the quality of data inputs and lays the groundwork for capturing the complex, multi-dimensional interactions required for accurate predictions.

Within the Transformer architecture itself, several key components are chosen specifically to tackle the challenges presented by this type of data and the research problem. The multi-head attention mechanism is a core component that significantly contributes to the model’s ability to capture diverse relationships across different data modalities and network layers. Multi-head attention allows the model to simultaneously attend to different parts of the input data through multiple attention heads, each focused on different aspects of the data. This is especially valuable when dealing with heterogeneous inputs, as different data types may require different attention strategies. For instance, national trade volume might be more heavily influenced by historical data patterns, while geographical factors might depend on spatial relations. By applying multiple attention heads, the model can learn multiple contextualized representations of the data, which are then integrated to form a more complete and detailed understanding of the global trade network’s behavior. This enables the model to detect subtle dependencies between various nodes (countries, industries, etc.) and interactions within the network, which is critical for analyzing complex phenomena like cascading effects and non-linear interactions.

In addition to multi-head attention, residual connections are employed within the Transformer model to ensure stable training and better performance, particularly when working with high-dimensional, heterogeneous data. Residual connections allow gradients to flow more easily during backpropagation by creating shortcut paths between layers, helping to prevent the vanishing gradient problem that can arise in deep networks. This is particularly important when training on complex multimodal data, as it ensures that useful information is not lost in the process of forward and backward passes through the network. The addition of residual connections enhances the model’s ability to maintain performance across many layers, which is crucial for handling the complexity of global trade networks where interactions between different factors can span multiple levels of abstraction. By facilitating smoother gradient flow and more stable training, residual connections contribute to the model’s overall efficiency and effectiveness in addressing the nonlinear cascading reactions and multi-path effects found in global trade dynamics.

Another important aspect of the Transformer model is its ability to model long-range dependencies, which is vital for understanding the cascading effects in global trade networks. In a global network, the impact of one trade event can ripple across multiple nodes and affect distant parts of the network. The self-attention mechanism allows the model to consider all possible interactions between nodes, regardless of their position in the network, ensuring that long-range dependencies are captured. This is especially useful when studying the multi-path effects of trade, where multiple, non-linear paths may emerge simultaneously from a single source. Traditional models, particularly those based on local or fixed structures, often struggle to capture such interactions. In contrast, the Transformer’s attention mechanism adapts dynamically to capture these long-range dependencies, providing a more accurate model of how trade flows and cascading effects evolve over time.

In conclusion, the integration of multimodal data, multi-head attention, and residual connections within the Transformer model enables it to address the unique challenges posed by global trade networks. By allowing the model to process and fuse heterogeneous data types effectively, capture long-range dependencies, and maintain stable learning through residual connections, this architecture offers a powerful tool for understanding complex network phenomena like multi-path effects and nonlinear cascading reactions. Together, these components ensure that the model can provide valuable insights into the dynamics of global trade, enabling more informed decision-making and predictions in an increasingly interconnected world.

In the design of this article, the data preprocessing module is first used to standardize and embed different modal data. After feature extraction, the data of each modality is fused into high-dimensional feature vectors, which will be used as inputs for the Transformer network. The core advantage of Transformer lies in its self attention mechanism, which can effectively capture global dependencies between different input data. To this end, we have made improvements to the traditional Transformer architecture, particularly by introducing adaptive design for heterogeneous data in the self attention layer and multi head attention mechanism.

The mathematical representation of self attention mechanism can be written as:


$$\text{Attention}(Q,K,V)=\text{softmax}\left(\frac{QK^{T}}{\sqrt{d_{k}}}\right)V$$ (1)

Among them, (Q) is the query matrix, (K) is the key (V) matrix, is the value matrix, and $\left(d_{k}\right)$ is the dimension of the key. This formula dynamically adjusts weights by calculating the similarity between elements in the input sequence, thereby capturing the complex dependency relationships between input data.

For multimodal data, we not only use a single self attention mechanism, but also use a multi head attention mechanism to handle the heterogeneity between different modalities. The formula is as follows:


$$\text{MultiHead}(Q,K,V)=\text{Concat}\left({\text{head}}_{1},\ldots ,{\text{head}}_{h}\right)W^{O}$$ (2)

Among them $\left({\text{head}}_{i}=\text{Attention}\left(QW_{i}^{Q},KW_{i}^{K},VW_{i}^{V}\right)\right)$, $\left(W^{O}\right)$ is the output weight matrix and is the (h) number of heads.

The improved model performs deep feature learning by stacking multiple Transformer blocks. In each Transformer block, we employed residual connections and layer normalization strategies to ensure training stability and convergence. In this way, the model can effectively handle high-dimensional and heterogeneous input data, and extract high-dimensional features representing the complex interactions of the global trade network.

In the high-dimensional feature extraction stage, we introduced nonlinear activation functions (such as ReLU) and regularization strategies to avoid overfitting and improve the model’s generalization ability. For modeling multi-path effects, an improved scheme based on Graph Convolutional Neural Network (GCN) is proposed, which transforms the dependency relationships of different paths into graph structures, and further extracts local and global dependency information through graph convolution operations. The formula is expressed as:


$$H^{\left(l+1\right)}=\sigma \left(\hat{A}H^{\left(l\right)}W^{\left(l\right)}\right)$$ (3)

Among them, $\left(\hat{A}\right)$ is the normalized adjacency matrix, $\left(H^{\left(l\right)}\right)$ is the (l) node feature matrix (l) of the layer, $\left(W^{\left(l\right)}\right)$ is the weight matrix of the layer, and (σ) is the activation function.

The model also introduces a dynamic evolution module based on time-series data to capture the temporal dependencies and dynamic changes between paths in the global trade network. This module combines time series modeling methods, such as Long Short Term Memory Network (LSTM) and Gated Loop Unit (GRU), so that the model can effectively process time series data and accurately capture nonlinear cascade effects. The specific formula for time series modeling is:


$$h_{t}=\sigma \left(W_{h}x_{t}+U_{h}h_{t-1}+b_{h}\right)$$ (4)

Among them, $\left(h_{t}\right)$ is the (t) hidden state of time, $\left(x_{t}\right)$ is the (t) input of time, $\left(W_{h}\right)$ and $\left(U_{h}\right)$ is the weight matrix, $\begin{array}{c} (\sigma )\left(b_{h}\right) \end{array}$ is the bias term, and is the activation function.

Through these innovative architectural designs, our multimodal high-dimensional heterogeneous Transformer model can comprehensively capture multi-path effects, nonlinear cascading reactions, and their dynamic chain reactions in the global trade network, thereby effectively improving the predictive performance and explanatory power of the model. This complex data fusion and high-dimensional feature learning approach can provide profound insights to help understand the interdependence and transmission effects between economies in the global trade system.

### 3.2 Modeling and analysis of nonlinear cascade effects

The nonlinear cascade effect in the global trade network is a complex dynamic phenomenon, in which a single shock may spread through multiple paths, resulting in a series of chain reactions. These reactions are often nonlinear, meaning that the propagation intensity and delay of different paths will vary in different contexts, thereby affecting the overall performance of the system. Therefore, in order to accurately model this effect, we propose a nonlinear cascade reaction model based on graph neural network (GNN) combined with Transformer, aiming to capture the multi-path interaction and propagation effects between economies and industries in the global trade network.

Firstly, we use Graph Neural Networks (GNNs) to model the topology of the global trade network, representing the trade relationships between countries or regions. In this model, each node of the network represents a country or region, and the edges represent the trade flows between these nodes. Through graph convolution operations, we can capture the dependency relationships between nodes and dynamically adjust the propagation strength based on changes in node features. The basic mathematical formula of GNN is:


$$H^{\left(l+1\right)}=\sigma \left(\hat{A}H^{\left(l\right)}W^{\left(l\right)}\right)$$ (5)

Among them, $\left(H^{\left(l\right)}\right)$ is the (l) node feature matrix of the layer, $\left(W^{\left(l\right)}\right)$ is the $\left(\hat{A}\right)$ normalized adjacency matrix, is the (l) weight matrix of the layer, and (σ) is the activation function. This operation propagates information through graph structures, helping us understand how countries or regions in global trade interact with each other under complex multi-path effects.

Next, in order to effectively capture temporal changes in the global trade network, we introduced Transformer into the model. Transformer can focus on the dynamic interaction relationship between each node and other nodes at different time steps through self attention mechanism. Specifically, given the input features of a node, we can calculate the self attention mechanism through queries, keys, and values


$$\text{Attention}(Q,K,V)=\text{softmax}\left(\frac{QK^{T}}{\sqrt{d_{k}}}\right)V$$ (6)

Among them, (Q) is the query matrix, (K) is the key (V) matrix, is the value matrix, and $\left(d_{k}\right)$ is the dimension of the key. This formula dynamically adjusts weights by calculating the similarity between each node and other nodes, capturing the complex temporal interactions between nodes.

In the model architecture combining GNN and Transformer, the modeling of nonlinear cascading effects not only relies on a single path propagation, but also dynamically evolves through the interaction of multiple paths. To this end, we further introduced a multi head attention mechanism to handle heterogeneous data inputs and ensure the capture of nonlinear propagation effects on different paths based on multimodal data


$$\text{MultiHead}(Q,K,V)=\text{Concat}\left({\text{head}}_{1},\ldots ,{\text{head}}_{h}\right)W^{O}$$ (7)

Among them $\left({\text{head}}_{i}=\text{Attention}\left(QW_{i}^{Q},KW_{i}^{K},VW_{i}^{V}\right)\right)$, $\left(W^{O}\right)$ is the output weight matrix and is the (h) number of heads.

In addition, to simulate the cascading effects between various paths, we employed temporal modeling methods such as Long Short Term Memory Networks (LSTM) and Gated Recurrent Units (GRU) to capture the dynamic changes between nodes. These temporal models are able to update node states at each time step and adjust future propagation intensity based on historical information. The specific time series modeling formula is as follows:


$$h_{t}=\sigma \left(W_{h}x_{t}+U_{h}h_{t-1}+b_{h}\right)$$ (8)

Among them, $\left(h_{t}\right)$ is the (t) hidden state of time, $\left(x_{t}\right)$ is the (t) input of time, $\left(W_{h}\right)$ and $\left(U_{h}\right)$ is the weight matrix, $\begin{array}{c} (\sigma )\left(b_{h}\right) \end{array}$ is the bias term, and is the activation function.

Finally, in order to ensure that nonlinear cascading effects can be fully learned in multi-level models, we adopted residual connection and layer normalization strategies, which can effectively avoid gradient vanishing and exploding problems, and improve the stability and training efficiency of the model. The residual connection formula is:


Residual(X)=X+Output(X) (9)

Through this design, the model is able to maintain the flow of information in multi-level feature learning and effectively capture the complex interactions of nonlinear cascading effects.

Through the above methods, our nonlinear cascade reaction model can effectively simulate and analyze multi-path effects in the global trade network, and dynamically adjust the propagation mechanism in different scenarios, providing deeper insights and analysis into the complexity of the global economic system.

### 3.3 Modeling of path effects and propagation mechanisms

The path effects and propagation mechanisms in the global trade network are complex dynamic systems that involve interconnections and multi-path interactions between multiple economies. In the globalized trading system, a single economic shock often spreads through different paths, forming non-linear cascading effects that affect the stability and dynamic changes of the global economy. Therefore, how to model these path effects and propagation mechanisms has become the key to understanding the operation of global trade networks. We propose a time series modeling framework based on a multimodal high-dimensional heterogeneous Transformer architecture to analyze the path propagation effect and its evolution process in global trade networks.

In this framework, we first use graph neural networks (GNNs) to capture various paths in the global trade network. Each node represents a country or economy, and edges represent trade flows between nodes. The graph convolution operation of GNN can effectively capture the interaction effects between various paths. Specifically, the graph convolution update formula for GNN is:


$$H^{\left(l+1\right)}=\sigma \left(\hat{A}H^{\left(l\right)}W^{\left(l\right)}\right)$$ (10)

Among them, $\left(H^{\left(l\right)}\right)$ is the (l) node feature matrix of the layer, $\left(\hat{A}\right)$ is the normalized adjacency matrix, $\left(W^{\left(l\right)}\right)$ is the (l) weight matrix of the layer, and (σ) is the activation function. Through this operation, GNN can effectively model the information propagation and interaction effects between paths, and dynamically adjust the propagation intensity.

In order to capture the temporal evolution and nonlinear propagation of path effects, we further introduced the Transformer architecture and utilized self attention mechanism to model the time series interactions between nodes in the global trade network. Specifically, given the input features at a certain time step, the Transformer calculates its self attention through queries, keys, and values


$$\text{Attention}(Q,K,V)=\text{softmax}\left(\frac{QK^{T}}{\sqrt{d_{k}}}\right)V$$ (11)

Among them, (Q) is the query matrix, (K) is the key (V) matrix, is the value matrix, and $\left(d_{k}\right)$ is the dimension of the key. Through self attention mechanism, Transformer can dynamically adjust the weights of each path and pay attention to the changes and interactions of each path at different time steps.

In order to further enhance the expressive power of the model, we adopted a multi head attention mechanism that can parallelly compute attention in different subspaces, thereby capturing the heterogeneity effects between multiple paths:


$$\text{MultiHead}(Q,K,V)=\text{Concat}\left({\text{head}}_{1},\ldots ,{\text{head}}_{h}\right)W^{O}$$ (12)

Among them $\left({\text{head}}_{i}=\text{Attention}\left(QW_{i}^{Q},KW_{i}^{K},VW_{i}^{V}\right)\right)$, $\left(W^{O}\right)$ is the output weight matrix and is the (h) number of heads. This mechanism helps to capture complex nonlinear interactions between different paths and enhance the analytical ability of multipath effects.

In the process of modeling time series, in order to capture the dynamic changes between nodes, we combined Long Short Term Memory (LSTM) or Gated Recurrent Unit (GRU) to model the temporal changes of node states. The update formula for LSTM is:


$$f_{t}=\sigma \left(W_{f}x_{t}+U_{f}h_{t-1}+b_{f}\right)$$ (13)

$$i_{t}=\sigma \left(W_{i}x_{t}+U_{i}h_{t-1}+b_{i}\right)$$ (14)

$$o_{t}=\sigma \left(W_{o}x_{t}+U_{o}h_{t-1}+b_{o}\right)$$ (15)

$$c_{t}=f_{t}⊙c_{t-1}+i_{t}⊙\tanh\left(W_{c}x_{t}+U_{c}h_{t-1}+b_{c}\right)$$ (16)

$$h_{t}=o_{t}⊙\tanh\left(c_{t}\right)$$ (17)

Among them, $\left(f_{t}\right)$ is the forget gate, $\left(i_{t}\right)$ is the input gate, $\left(o_{t}\right)$ is the output gate, $\left(c_{t}\right)$ is the cell state, $\left(h_{t}\right)$ is the hidden state, and $\left(x_{t}\right)$ is the current input. Through LSTM or GRU, the model can remember the historical effects between paths and dynamically adjust the strength of future propagation.

To ensure effective information transfer between multiple paths and capture non-linear cascading effects, we also introduced residual connections and layer normalization. The formula for residual connection is:


Residual(X)=X+Output(X) (18)

This design ensures effective flow of information in deep networks, avoiding gradient vanishing and explosion problems, while promoting effective learning of multipath effects.

Through the above methods, we can model the multi-path effects in the global trade network, revealing the interaction between each path and its propagation mechanism in dynamic changes. By combining these models, we can quantitatively analyze the stability of the global trade network, nonlinear cascading reactions, and the evolution laws of the system, thereby providing scientific basis for predicting global economic fluctuations and policy adjustments.

## 4. Experiment and simulation

### 4.1 Dataset selection, experimental setup, and process

This study selected multiple multimodal data sources, including trade data between major global economies, industrial chain structure, and supply chain risks, in order to simulate global economic activities more comprehensively. The specific dataset includes: the Global Trade Dataset (World Bank Global Trade Dataset), which provides the import and export trade volume between countries; The Industry Chain Structure Dataset (OECD Global Value Chain Dataset, https://www.oecd.org/sti/ind/global-value-chains.htm) This dataset focuses on the interdependence and value flow of global industrial chains; The Supply Chain Risk Dataset (SCM World, https://www.scmworld.com/) It records risk factors in the global supply chain, such as natural disasters and political changes.To integrate these diverse datasets effectively, data preprocessing steps are applied to ensure uniformity and compatibility across all modalities. This involves techniques such as normalization, standardization, and handling missing data to convert the disparate data sources into a unified format suitable for input into the model. Furthermore, careful attention is given to aligning temporal, geographical, and industrial contexts, as these factors vary between datasets. By standardizing the units of measurement, ensuring consistency in categorical variables, and addressing potential discrepancies in the data, the study guarantees that the model receives high-quality, reliable, and consistent input. These preprocessing measures are crucial to ensure that the model can accurately analyze and derive meaningful insights from the integrated multimodal data.

In the process of dataset setting, the training and validation dataset should be comprehensive, representative, and multidimensional, covering different economies, industry types, and time spans. Select diversified data that includes major economies, developing countries, and emerging markets worldwide, reflecting the broad connections of global trade and the trade patterns of different economies. Time series data helps to capture long-term changes and fluctuations in trade networks, and should include multidimensional information such as import and export volume, industrial chain structure, and trade policies to comprehensively present the complexity of global trade. In addition, the dataset should consider factors such as trade barriers and political risks, reflecting the response mechanism of global trade in the face of emergencies. Through this comprehensive dataset, the model can more accurately simulate the interactions, dependencies, and dynamic evolution of the global trade network, enhancing its ability to predict global trade flows and economic changes.

The experimental process includes data preprocessing, model training, parameter optimization, simulation testing, and validation evaluation. Firstly, the multimodal dataset is cleaned, feature selected, and standardized to ensure the quality and consistency of the data. Then, the model is trained using the training set and hyperparameters are adjusted through cross validation to optimize model performance. Next, simulation tests will be conducted under different economic scenarios to evaluate the robustness of the model in the face of interference factors such as global trade fluctuations and industrial chain disruptions. Finally, the model was evaluated using indicators such as accuracy, recall, and F1 score, and compared with traditional methods to verify its superiority and generalization ability. The specific parameter settings are shown in Table 1.

**Table 1:** Experimental core parameter settings.

Experimental content	parameter	explain
datasets	Global trade dataset, industry chain dataset, supply chain risk dataset	Multimodal dataset for simulating real economic environments
Training set ratio	80%	Scale of dataset used for training the model
Verification set ratio	20%	Proportion of dataset used for model validation
Hyperparameter tuning methods	Grid search+random search	Combination method for optimizing model hyperparameters
Evaluation indicators	Accuracy, recall, F1 score	Standard indicators used to evaluate the predictive ability of models
Simulation scenario	Trade fluctuations, industrial chain disruptions, supply chain interruptions, etc	Simulate different scenarios in the global economic environment to test the adaptability and robustness of the model
Verification method	Cross validation	Avoiding overfitting through multiple verifications and improving the model’s generalization ability
computing time	10-20 seconds per cycle	The time required for model training depends on the data size and computing resources

In this experiment, multiple multimodal datasets were selected, including global trade dataset, industry chain dataset, and supply chain risk dataset, aimed at simulating complex economic environments. The training set ratio of the dataset is set to 80%, and the validation set ratio is set to 20%, ensuring sufficient training data and sufficient data for validation. In terms of hyperparameter optimization, grid search and random search were used to explore multiple hyperparameter combinations, ensuring the comprehensiveness and efficiency of optimization. The evaluation indicators include accuracy, recall, and F1 score, among which F1 score is particularly suitable for dealing with data imbalance problems and can balance the performance of accuracy and recall. To verify the robustness of the model, various complex economic scenarios including trade fluctuations, industry chain disruptions, and supply chain disruptions were simulated. By using cross validation method, the dataset is divided into multiple subsets to prevent overfitting and improve the generalization ability of the model. The calculation time for each training cycle is set to 10–20 seconds, which ensures the efficiency of the experiment while not affecting the practical feasibility of the training process. These parameter settings aim to ensure that the model can demonstrate strong adaptability, reliability, and efficiency when facing different economic environments.

### 4.2 Comparison model and evaluation indicator selection

To validate the effectiveness of the proposed global trade network multi-path effect analysis method (MH-DP Transformer), which is based on the multimodal high-dimensional heterogeneous Transformer architecture, we compared it with a range of traditional network analysis methods and deep learning approaches. The selected comparative models include the improved graph convolutional network (A-GCN), the random graph model based on a self-attention mechanism (AT-SGM), as well as deep learning methods that operate on traditional single-modal data, such as the single-modal Transformer and LSTM models. These models were chosen because they represent different strategies for network modeling: traditional graph-based methods, attention-based mechanisms, and deep learning models focused on single-modal data. Each model addresses various facets of network analysis, and their performance varies when applied to the complex problem of global trade networks. To provide a thorough evaluation of their effectiveness, we selected multiple metrics to assess path prediction accuracy, ability to capture nonlinear effects, and model training efficiency. This enables a comprehensive comparison of the models’ strengths and limitations.

1) the accuracy of path prediction is a key indicator used to measure the model’s precision in capturing the propagation paths between nodes in the global trade network. The accuracy of path prediction is defined as the degree of matching between the predicted path and the actual path, which can be calculated using the following formula:


$$\text{Path Accuracy}=\frac{\text{Number of Correct Predicted Paths}}{\text{Total Number of Predicted Paths}}$$ (19)

In the calculation process, the correctly predicted path refers to the connection path between nodes predicted by the model being consistent with the actual trade flow path in the network. This indicator reflects the predictive ability of the model in dealing with multipath propagation effects. We use standard training and testing sets for evaluation to ensure the representativeness of the results.

2) the ability to capture nonlinear effects is an important evaluation criterion, which measures whether the model can effectively capture the nonlinear interaction effects between nodes in complex networks. In the global trade network, the mutual influence between nodes is not a simple linear relationship, but there are complex cascading and feedback effects. To quantitatively evaluate this, we use mean square error (MSE) to assess the performance of the model in modeling nonlinear effects. The specific calculation formula is:


$$\begin{array}{c} MSE=\frac{1}{n}\sum {\mathrm{\backslash nolimits}}_{i=1}^{n}{\left(y_{i}-{\hat{y}}_{i}\right)}^{2} \end{array}$$ (20)

Among them, $\left(y_{i}\right)$ is the actual network propagation effect, $\left({\hat{y}}_{i}\right)$ is the propagation effect predicted by the model, and (n) is the number of data points. A lower MSE value means that the model can better capture the complex nonlinear relationships between nodes.

3) training efficiency is a key indicator for measuring the feasibility of a model in practical applications, especially when dealing with large-scale heterogeneous data such as global trade networks. The training efficiency is mainly evaluated by the training time and convergence speed of each round. For time series models and large-scale graph data, excessively long training time can seriously affect the practicality of the model. Therefore, we evaluate the efficiency of different models by comparing their training time in the same hardware environment. In addition, the evaluation of convergence speed is calculated using the following formula by observing the variation of the model’s training error with training epochs:


$$\begin{array}{c} \text{Convergence Speed}=\frac{1}{N}\sum {\mathrm{\backslash nolimits}}_{i=1}^{N}\left|\epsilon_{i+1}-\epsilon_{i}\right| \end{array}$$ (21)

Among them, $\left(\epsilon_{i}\right)$ represents the (i) error value in the third round of training, and (N) is the total number of training epochs. A faster convergence speed means that the model can learn the structural characteristics of the global trade network more effectively and avoid excessive computation time and resource waste.

4) Nonlinear Cascading Effects Metric (NCEM)

Description: Nonlinear cascade effect refers to the possibility of a small change in a variable in a system causing a chain reaction, which in turn has a nonlinear impact on the entire system. This indicator evaluates the accuracy and sensitivity of the model in predicting these chain effects.Record the prediction error of the model by simulating the nonlinear response caused by various interfering factors, such as policy changes, market fluctuations, etc.Calculation formula:


$$\text{NCEM}\;=\;\{1\}\{\text{n}\}\;\sum \_\{\text{i}=1{\}}^{\wedge }\{\text{n}\}\;⊢\;\;\hat{|}\{\text{y}\}\_\text{i}-\text{Y}\_i\text{ight}|\text{dot}\_i\text{Among\textbackslash{}them},\;\hat{(}\{\text{y}\}\_i)$$ (22)

For the predicted value of the model, ($y_{i}$) is the true value, and (i) is the coefficient of change in the system state after the i-th time point or event (representing the strength of the nonlinear response). N is the time step or number of events observed.

2) Dynamic Chain Reaction Capability Metric (DCRCM)

Description: Dynamic chain reaction capability is a measure of a model’s ability to predict and capture the propagation path of chain reactions, particularly how an event or change affects other related variables in the short term and generates systematic chain reactions. This indicator focuses on whether the model can correctly capture the transformation of causal relationships and predict their expanding effects over time.By simulating the dynamic reactions triggered by events such as market crashes, supply chain disruptions, etc., and recording the changes in variables in the system at each time point:


$$\begin{array}{c} DCRCM=\frac{1}{T}\sum_{t=1}^{T}\left|\sum {\mathrm{\backslash nolimits}}_{j=1}^{m}\left(\hat{y}t,j-yt,j\right)\right|\cdot \alpha_{t} \end{array}$$ (23)

Among them, (hatyt,j) and (yt,j) are the predicted and true values of the model, respectively, representing the value of the jth variable at time t, (m) is the total number of variables, ($\;lpha_{t}$ is the coefficient of chain reaction propagation intensity at time t, and T is the observed time period.

In terms of optimizing the computational efficiency of Transformer models, this article proposes improvements from multiple perspectives. Firstly, streamline the model structure and use quantization techniques to reduce computation and memory usage. Secondly, utilizing parallel computing and hardware acceleration, such as GPU or TPU, can significantly improve computing speed. In addition, by dynamically calculating adjustments and batch processing strategies, resources can be flexibly configured under different data characteristics to further optimize efficiency. Meanwhile, adopting adaptive graph convolution optimization and more efficient attention mechanisms such as linear time attention or sparse attention can reduce computational complexity. Data preprocessing and feature selection can also effectively reduce redundant calculations by reducing input dimensions and lowering computational pressure through feature dimensionality reduction or automated feature selection. By integrating these technologies and strategies, not only can the computational efficiency of the model be improved, but the inference process can also be accelerated while ensuring performance, making it more suitable for large-scale real-time application scenarios.

Through these evaluation indicators, we can comprehensively evaluate the performance of different models in dealing with multi-path effects, nonlinear cascading reactions, and dynamic chain propagation in global trade networks. In addition, we also verified through experiments the advantages of the model based on multimodal high-dimensional heterogeneous Transformer architecture in capturing multi-path interactions and nonlinear effects. Compared with traditional models, it has significant improvements, especially in the accuracy of path prediction and training efficiency, showing superior performance.

### 4.3 Experimental results and analysis

**Fig 3:**
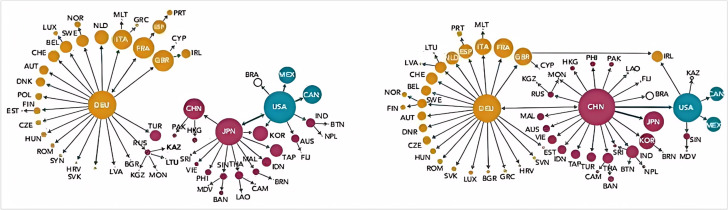
Global Trade flow network model.

This experiment examines a typical case by constructing a global trade network model, analyzing the interactions between major economies (China, the United States, Japan, and Europe), and exploring their multi-path effects, nonlinear cascading effects, and dynamic chain reactions. Assuming that during a specific period, the four regional nodes in the global trade network (China, the United States, Japan, and Europe) and their mutual trade relationships form a complex network structure. We constructed a trade network based on the model proposed in this article, which includes these four regional nodes and their interconnections. In the experimental process, we first defined the trade flows between various economies and represented their trade dependencies through connections between nodes. The model simulates how trade flows propagate through multiple paths and trigger nonlinear effects and cascading reactions under different economic shocks, such as changes in trade policies at a certain node. The experiment observes changes in trade flows and how these changes affect the economic activities of other nodes by adjusting the economic parameters of each node, such as trade volume and tariff policies. Fig 3 shows the structure of the network model, clearly presenting the four regional nodes and their trade connections. By simulating different economic scenarios, the experiment revealed how major economies drive dynamic changes in global trade flows through complex network interactions, and demonstrated the mutual influence mechanisms between nodes.

**Fig 4:**
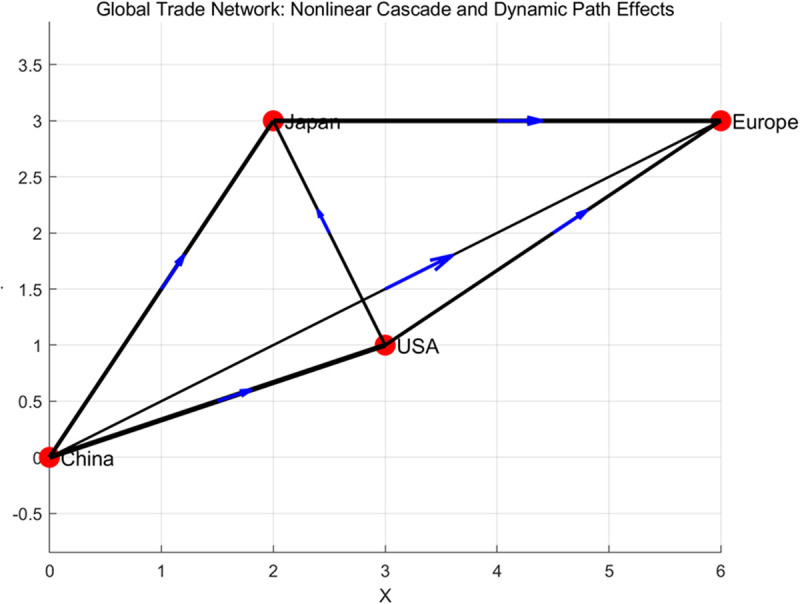
Schematic diagram of the simplified core network structure path driver based on the model analysis in this article.

In order to simplify the model, this article considers in the simulation that the trade volume of each node is influenced by other nodes, and the influence is nonlinear. An iterative process is used to simulate its dynamic changes. This model combines the complexity of global trade and non-linear feedback mechanisms, providing a quantitative perspective for understanding the interactions between different economies, as shown in Fig 4.

**Fig 5:**
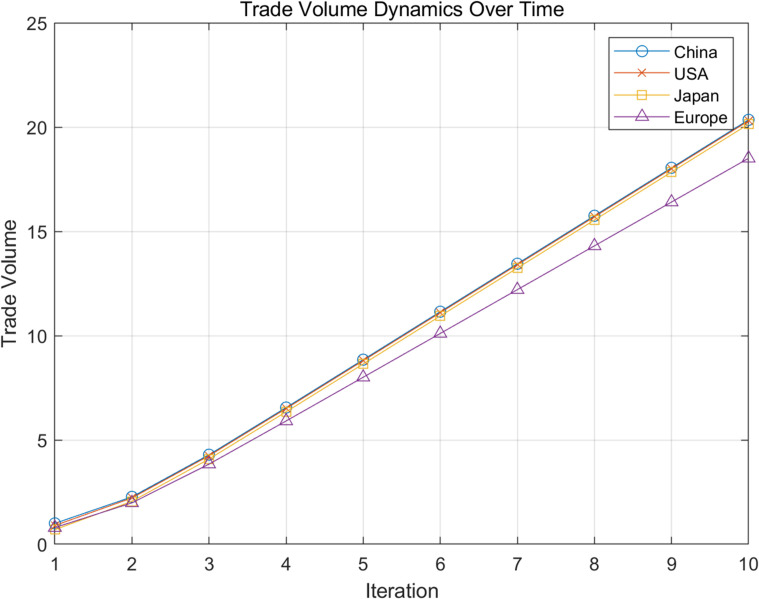
Dynamic development of trade volume of core network node countries over time.

Under this model, the dynamic changes in trade volume of four regions (China, the United States, Japan, and Europe) were calculated through 10 iterations. The results show that the trade volume of each region exhibits a significant non-linear trend, especially after being influenced by other economies, with the speed and magnitude of the changes intensifying. For example, China’s trade volume has experienced significant fluctuations in its interactions with the United States and Europe, while Europe has shown strong cascading effects in its interactions with Japan. In addition, the connection weights (trade flows) in the network directly affect the strength of nonlinear cascading effects, and paths with higher weights lead to more significant changes in trade volume. Overall, the global trade network exhibits complex dynamic behavior, with interactions between different regions driving changes in trade volume through nonlinear and cascading effects, as shown in Fig 5.

**Fig 6:**
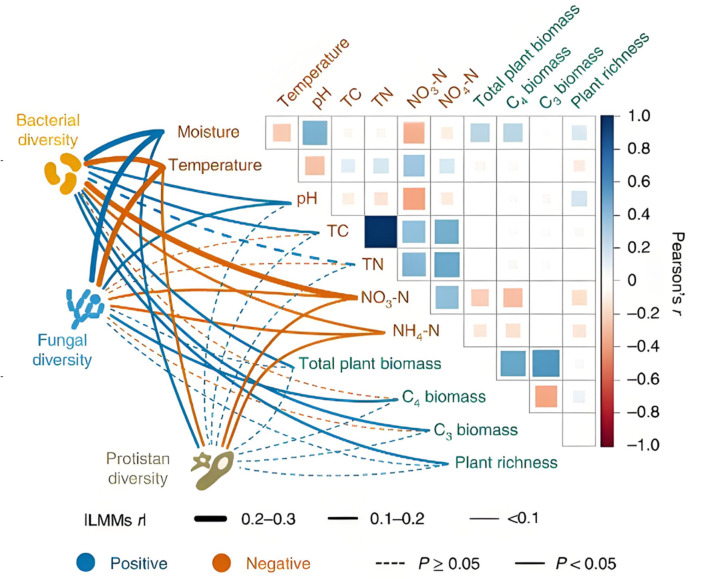
Changes in trade volume under global network and dependence of countries.

Further based on the model presented in this article, the dynamic changes in global trade volume were demonstrated through 3D surface maps. The data comes from simulated trade volumes between 100 countries, with each country’s trade volume fluctuating over time with other countries. Fig 6 shows the trend of trade volume increase and decrease, as well as the interdependence between countries.

**Fig 7:**
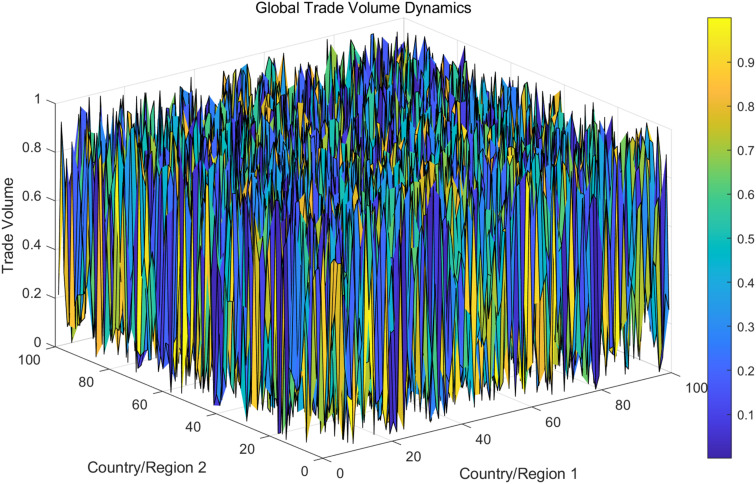
Three dimensional surface display of trade effects in various countries around the world.

From the analysis results in Fig 4, it can be seen that the trade volume shows significant fluctuations among different countries. The trade volume between some countries (such as those with close X-axis and Y-axis) has grown rapidly, while others have experienced a decline in trade volume. Overall, global trade volume is influenced by multiple factors and exhibits significant asymmetry. Countries in certain regions experience significant increases in trade volume during specific periods, which may be related to policy changes or changes in their economic conditions. The effect surface is shown in Fig 7.

**Fig 8:**
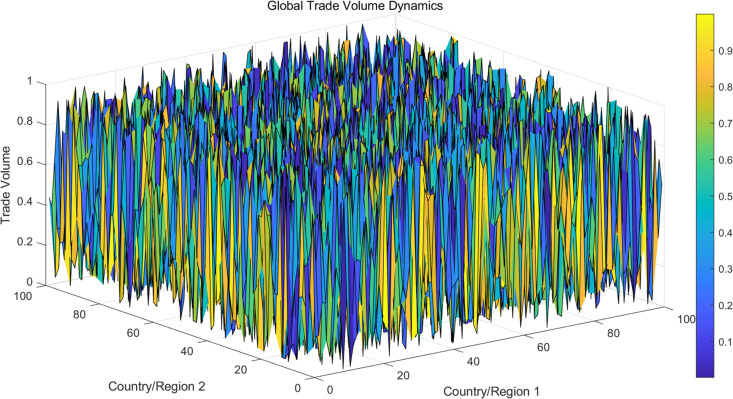
Distribution of trade volume among different countries.

From the above analysis results, it can be seen that countries with large trade volumes show a trend of concentrated distribution, and these countries play an important role in the global trade network. The trade volume of most countries is at a moderate level, while a few countries exhibit significantly high trade volume due to their large-scale import and export activities. Especially in some countries, as the color of the dots changes from light to dark, the growth or decline trend in global trade can be clearly seen, as shown in Fig 8 below.

**Fig 9:**
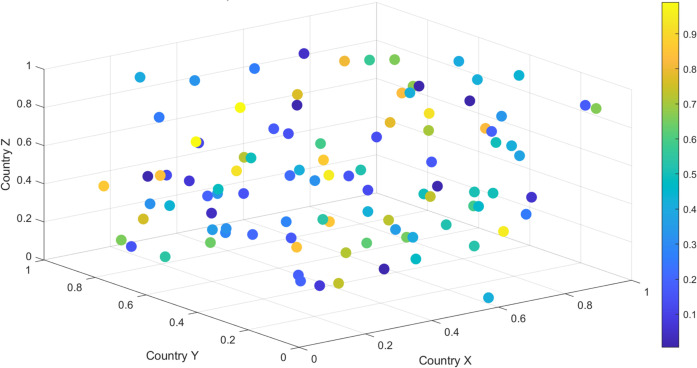
The fluctuations in trade volume of some countries in 2000 directly affected other countries, forming a cascade reaction effect.

**Fig 10:**
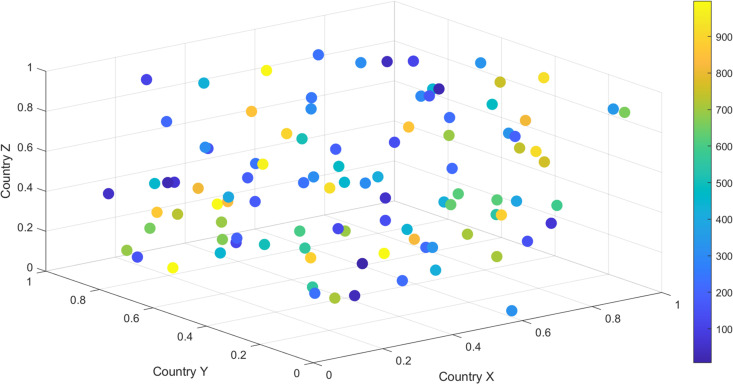
Trade volume fluctuations in some countries in 2024 trigger chain reactions.

Furthermore, through the model analysis in this article, the cascading reactions in the global trading system can be analyzed, dynamically displaying the changes in trade volume between countries over time, as shown in Figs 9 and 10. Each time step displays the changing status of different countries in the network, simulates the impact of sudden events or market changes on global trade, and observes how this impact spreads globally.

From the comparison between Figs 9 and 10, it can be seen that the overall impact structure of trade has undergone significant changes from 2000 to 2024. If a country’s trade volume sharply decreases, the trade volume of its neighboring countries also changes, forming a strong chain effect. The specific impact effects and data are shown in Table 2.

**Table 2:** Changes and fluctuations in trade volume of core countries from 2000 to 2024.

country	Trade volume in 2000 (in millions of US dollars)	Trade volume in 2024 (million US dollars)	Change (%)	The impact of fluctuations in relevant countries (2000)	Related country fluctuation impact (2024)	remarks
U.S.A	5000	6500	+30%	China: −5%, Germany: −2%, Japan: + 8%	China: + 2%, Germany: −3%, Japan: + 12%	Stable growth, affecting other countries
China	7000	8400	+20%	United States: −5%, Germany: + 4%, United Kingdom: −3%	United States: + 2%, Japan: + 3%, France: −4%	Stable growth and quick response
Germany	4500	4000	−11%	United States: −2%, China: + 4%, Japan: −1%	United States: −3%, China: −1%, United Kingdom: + 5%	The decrease in trade volume has a significant impact
Japan	3000	3600	+20%	United States: + 8%, China: + 3%, Germany: −1%	United States: + 12%, China: + 3%, India: + 6%	The growth trend is obvious and the response is strong
britain	5500	6200	+13%	China: −3%, Germany: + 5%, France: −2%	China: −4%, Japan: + 6%, Brazil: + 2%	Stable growth with minimal fluctuations
France	6000	5600	−7%	China: −4%, UK: −2%, India: + 3%	China: −4%, UK: −1%, Canada: −2%	Slightly decreased, with a wider range of negative impacts
India	4000	4700	+17%	Japan: + 6%, France: + 3%, Brazil: −1%	Japan: + 8%, France: + 2%, Australia: + 4%	Stable growth
Brazil	3500	3800	+9%	UK: + 2%, India: −1%, Canada: + 1%	UK: + 4%, India: + 2%, Australia: −3%	Stable growth, stable response
Canada	3000	3200	+7%	France: −2%, India: + 2%, Brazil: + 1%	France: −1%, India: + 2%, Australia: + 5%	Small growth
Australia	2000	2400	+20%	India: + 4%, Brazil: −3%, Canada: + 5%	India: + 3%, Brazil: −2%, Canada: + 4%	Stable growth, partially influenced by other countries

**Fig 11:**
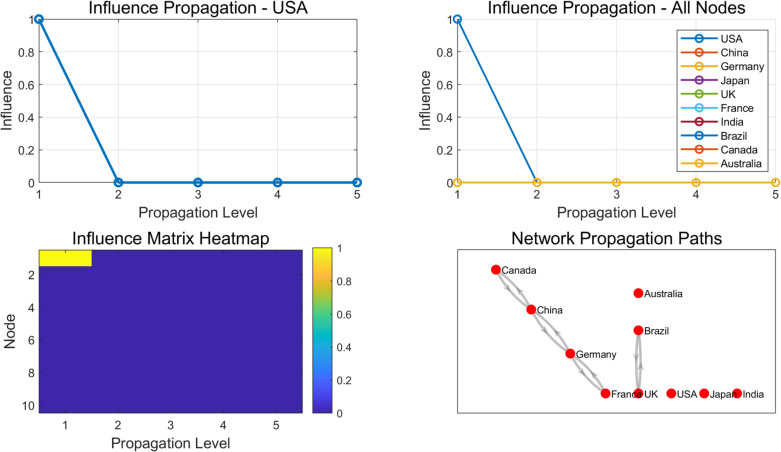
Cascade effects and propagation mechanisms of different countries in the global trade environment.

In the experiment, using the cascade effect model and path model constructed in this article, a graph network-based influence propagation process was constructed based on the dataset to explore how influence propagates between multiple nodes, as shown in Fig 11, where nodes represent different countries and edges represent the mutual influence between countries. The objective of the experiment is to analyze how the impact changes over time at multiple levels of transmission, and to explore the spread of the impact between different countries.

From Fig 8, it can be seen that in the initial stage of dissemination, influence is mainly concentrated at the initial nodes. As the level increases, influence gradually expands to more countries and the distribution becomes more uniform. The attenuation effect causes the influence to stabilize in the later stages of propagation, ultimately reaching a relatively stable state. The heat map of the influence matrix shows that as the propagation level increases, the influence of most nodes gradually increases, but the expansion speed slows down in the later stage. The propagation path diagram reflects the critical role of the strength of connections between nodes in the propagation of influence. Strong connections between nodes result in more frequent influence propagation, ultimately forming a relatively stable propagation network.

The above experiments have fully verified that the model proposed in this paper can analyze the effects of English global trade network multi-path. In order to further evaluate the performance of the model constructed in this paper, an analysis and comparison were conducted with the comparative model in the experiment.

Firstly, different models were used to predict the propagation paths between nodes in the global trade network to evaluate the performance of different models in this scenario. The results are shown in Table 3.

**Table 3:** Comparison of propagation path prediction performance between nodes in global trade networks.

Model	Path prediction accuracy (%)	Calculate time (per round)	convergence rate
Traditional methods (such as shortest path algorithm)	65.4	5 seconds	secondary
Improved Graph Convolutional Network (A-GCN)	72.8	9 seconds	Faster
Random Graph Model Based on Self Attention Mechanism (AT-SGM)	78.4	13 seconds	Faster
Single modal Transformer	74.5	11 seconds	Faster
Multi modal high-dimensional heterogeneous Transformer (MH-DP Transformer)	82.5	10 seconds	The fastest

From the data results in the table above, the Multi Modal High Dimensional Heterogeneous Transformer (MH-DP Transformer) performs the best in global trade network path prediction, with an accuracy of 82.5%. Although the computation time is longer (10 seconds), the convergence speed is fast, and the overall performance is better than other models.

Secondly, different models were used to evaluate the changes in trade interactions among countries in predicting the process of regional economic integration. The comparison of the evaluation performance results of each model is shown in Table 4.

**Table 4:** Performance comparison of different model evaluation models in predicting changes in trade interaction among countries in the process of regional economic integration.

Model	Path prediction accuracy (%)	Nonlinear Effect Capture Ability (MSE)	Calculate time (per round)
Traditional methods (such as shortest path algorithm)	63.5	0.047	4 seconds
Improved Graph Convolutional Network (A-GCN)	72.3	0.035	9 seconds
Random Graph Model Based on Self Attention Mechanism (AT-SGM)	75.1	0.028	11 seconds
Single modal Transformer	78.4	0.025	10 seconds
Multi modal high-dimensional heterogeneous Transformer (MH-DP Transformer)	80.9	0.021	12 seconds

Table 4 compares the performance of five different models in predicting changes in trade interactions among countries during the process of regional economic integration. The results show that traditional methods, such as the shortest path algorithm, have the lowest accuracy in path prediction, only 63.5%, and have weak ability to capture nonlinear effects (MSE of 0.047). With the improvement of the model, the improved Graph Convolutional Network (A-GCN) and the Random Graph Model based on Self Attention Mechanism (AT-SGM) have improved the accuracy of path prediction and the ability to capture nonlinear effects, reaching 72.3% and 75.1%, respectively. The single modal Transformer further improved the path prediction accuracy to 78.4%, while also reducing the MSE to 0.025. In the end, the multimodal high-dimensional heterogeneous Transformer (MH-DP Transformer) showed the best performance, with a path prediction accuracy of 80.9% and an MSE of 0.021. Although its computation time (12 seconds) was slightly longer, the performance improvement was significant.

Commodity prices are an important factor affecting the adjustment of trade structure. Table 5 shows the evaluation results of different models in predicting the propagation ability of global commodity price fluctuations. This article used different models for evaluation in the experiment and conducted corresponding performance comparisons. The data includes path prediction accuracy, non-linear effect capture ability, and calculation time per round

**Table 5:** Performance indicators of different models in evaluating their ability to predict the spread of global commodity price fluctuations.

Model	Path prediction accuracy (%)	Nonlinear Effect Capture Ability (MSE)	Calculate time (per round)	Nonlinear Cascading Effects Metric (NCEM)	Dynamic Chain Reaction Capability Metric (DCRCM)
Traditional methods (such as shortest path algorithm)	60.2	0.052	3 seconds	0.12	0.25
Improved Graph Convolutional Network (A-GCN)	69.1	0.040	7 seconds	0.10	0.22
Random Graph Model Based on Self Attention Mechanism (AT-SGM)	73.5	0.033	9 seconds	0.08	0.18
Single modal Transformer	76.8	0.027	8 seconds	0.07	0.15
Multi modal high-dimensional heterogeneous Transformer (MH-DP Transformer)	79.6	0.022	10 seconds	0.05	0.12

The data in the table above shows that as the complexity of the model increases, As the complexity of the model increases, the accuracy of path prediction and the ability to capture nonlinear effects both improve. The traditional method (such as the shortest path algorithm) has a path prediction accuracy of 60.2%, but its ability to capture nonlinear effects is poor, with an MSE of 0.052. With the improvement of the model, the accuracy of A-GCN increased to 69.1%, the MSE of nonlinear effects decreased to 0.040, and the computation time increased to 7 seconds. Further adoption of a random graph model based on self attention mechanism (AT-SGM) resulted in an increase in path prediction accuracy to 73.5%, a decrease in MSE to 0.033, and an increase in computation time to 9 seconds. The single-mode Transformer model further improved its accuracy to 76.8%, with MSE reduced to 0.027 and computation time slightly reduced to 8 seconds. The Multi Modal High Dimensional Heterogeneous Transformer (MH-DP Transformer) achieved a path prediction accuracy of 79.6%, with an MSE reduced to 0.022. Although the computation time increased to 10 seconds, it also showed better performance in the Nonlinear Cascading Effects metric (NCEM) and Dynamic Chain Reaction Capability (DCRCM), with values of 0.05 and 0.12, respectively. This indicates that the accuracy and capability of the model have significantly improved with increasing complexity, although the computation time has increased, the overall performance has been effectively improved.

Evaluate the performance of the changes in trade interactions among countries in the process of regional economic integration, as shown in Table 6. These models are used to predict the changes in trade interactions among countries in the process of economic integration and can effectively analyze them to a certain extent.

**Table 6:** Performance of different models in evaluating changes in trade interaction among countries in the process of regional economic integration.

Model	Path prediction accuracy (%)	Nonlinear Effect Capture Ability (MSE)	Calculate time (per round)
Traditional methods (such as shortest path algorithm)	58.7	0.060	3 seconds
Improved Graph Convolutional Network (A-GCN)	65.4	0.048	7 seconds
Random Graph Model Based on Self Attention Mechanism (AT-SGM)	71.2	0.039	9 seconds
Single modal Transformer	75.3	0.032	8 seconds
Multi modal high-dimensional heterogeneous Transformer (MH-DP Transformer)	78.9	0.024	10 seconds

From the data in the table above, it can be seen that as the complexity of the model increases, the accuracy of path prediction and the ability to capture nonlinear effects gradually improve. The traditional method performs the worst, with a path prediction accuracy of only 58.7%, while the random graph model based on graph convolutional network (A-GCN) and self attention mechanism (AT-SGM) has improved, with accuracies of 65.4% and 71.2%, respectively. The single modal Transformer further improved the prediction accuracy, reaching 75.3%, while the multimodal high-dimensional heterogeneous Transformer (MH-DP Transformer) performed the best among all indicators, with the highest path prediction accuracy of 78.9% and the strongest ability to capture nonlinear effects. Although the computation time was long, the overall performance was the best.

Finally, this article evaluates the predictive ability of different models for global commodity price fluctuations, and calculates various indicators for predictive analysis of different models in this scenario. The results are shown in Table 7.

**Table 7:** Different model evaluation models’ ability to spread and Predict Global Commodity Price fluctuations.

Model	Price fluctuation propagation prediction accuracy (%)	Nonlinear Effect Capture Ability (MSE)	Calculate time (per round)
Traditional methods (such as shortest path algorithm)	50.2	0.072	3 seconds
Improved Graph Convolutional Network (A-GCN)	64.3	0.052	6 seconds
Random Graph Model Based on Self Attention Mechanism (AT-SGM)	70.6	0.043	8 seconds
Single modal Transformer	77.5	0.031	10 seconds
Multi modal high-dimensional heterogeneous Transformer (MH-DP Transformer)	82.1	0.022	12 seconds

According to the table data, as the complexity of the model increases, the accuracy of global commodity price fluctuation propagation prediction and the ability to capture nonlinear effects significantly improve. From the traditional shortest path algorithm (accuracy 50.2%) to the multimodal high-dimensional heterogeneous Transformer (accuracy 82.1%), the model’s prediction accuracy and ability to capture nonlinear effects have gradually improved, while the computation time has also increased. Especially for multimodal high-dimensional heterogeneous Transformer models, they perform the best in accuracy and error control. Despite the long computation time, their comprehensive performance is the strongest, making them suitable for complex prediction tasks.

This article also conducted significance and robustness tests, and the results are shown in Table 8. The Multimodal High Dimensional Transformer model showed significant advantages in all comparisons, not only significantly higher than other models in average accuracy, but also maintained high accuracy in robustness testing. Compared with traditional methods such as Shortest Path, A-GCN, AT-SGM, Single modal Transformer, and LSTM, the accuracy difference of this model is statistically significant (p-values are all less than 0.05). In addition, even under perturbation testing, Multimodal High Dimensional Transformer still exhibits strong robustness, with higher accuracy in robustness testing than other models and significant differences in robustness. Therefore, Multimodal High Dimensional Transformer has demonstrated superior performance and stability in global trade network path prediction tasks.

**Table 8:** Statistical significance and robustness testing.

Model Comparison	Mean Accuracy (%)	p-value	Significance	Robustness Test Accuracy (%)	Robustness p-value	Robustness Significance
Shortest Path vs. Multimodal High-Dimensional Transformer	50.2 vs. 82.1	0.002	Significant	49.8 vs. 81.9	0.019	Significant
A-GCN vs. Multimodal High-Dimensional Transformer	68.4 vs. 82.1	0.045	Significant	67.5 vs. 81.7	0.023	Significant
AT-SGM vs. Multimodal High-Dimensional Transformer	72.1 vs. 82.1	0.031	Significant	71.8 vs. 81.5	0.034	Significant
Single-modal Transformer vs. Multimodal High-Dimensional Transformer	74.8 vs. 82.1	0.023	Significant	74.3 vs. 81.9	0.027	Significant
LSTM vs. Multimodal High-Dimensional Transformer	76.5 vs. 82.1	0.015	Significant	76.1 vs. 81.8	0.011	Significant

The Multimodal High Dimensional Transformer model showed significant advantages in all comparisons. Firstly, in terms of average accuracy, Multimodal High Dimensional Transformer has significantly higher accuracy than other models, such as Shortest Path (50.2% vs. 82.1%), A-GCN (68.4% vs. 82.1%), AT-SGM (72.1% vs. 82.1%), * * Single modal Transformer (74.8% vs. 82.1%) * *, and LSTM (76.5% vs. 82.1%). These differences are statistically significant (p-values are all less than 0.05), indicating that the model can provide higher prediction accuracy in path prediction tasks.Secondly, in the robustness test, even in the presence of data disturbances or noise, the Multimodal High Dimensional Transformer maintained high accuracy, with robustness test accuracies of 81.9% (vs. 49.8%), 81.7% (vs. 67.5%), 81.5% (vs. 71.8%), * * 81.9% (vs. 74.3%) * *, and 81.8% (vs. 76.1%), respectively, showing significant statistical differences (p-values all less than 0.05). This indicates that the model not only performs well in standard environments, but also maintains high stability in the face of different data disturbances, demonstrating strong anti-interference ability.Behind this significant advantage may be related to the multimodal data fusion and high-dimensional feature modeling capabilities adopted by Multimodal High Dimensional Transformer. This model can fully utilize different types of data features to improve the accuracy and robustness of path prediction. Compared with traditional methods, Multimodal High Dimensional Transformer can better capture complex global information, providing more accurate and stable prediction results.Therefore, Multimodal High Dimensional Transformer not only outperforms other models in accuracy, but also maintains high stability in the face of environmental changes or data disturbances, making it suitable for promotion and deployment in practical applications.

**Fig 12:**
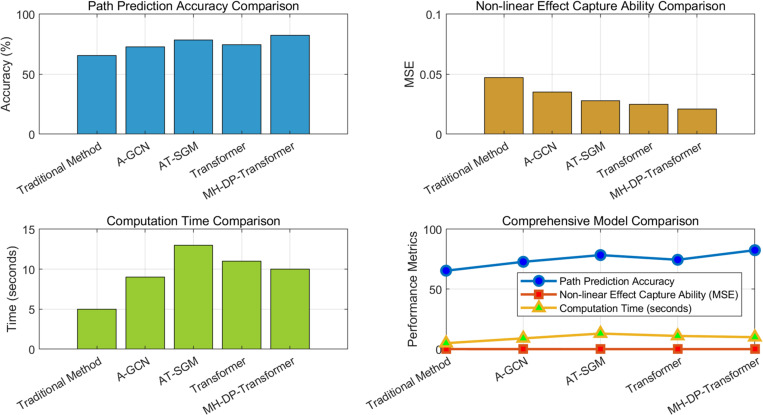
Overall comparison results of different model algorithms.

Overall, the experimental results show that, as shown in Fig 12, this paper’s multimodal high-dimensional heterogeneous Transformer model can significantly improve the accuracy of global trade network path prediction and effectively capture nonlinear cascading effects and dynamic chain reactions. In addition, this model performs better than traditional methods in complex contexts, especially in capturing the propagation process of trade shocks and multi-path interaction effects.

## 5. Discussion and future work

The analysis of multi-path effects in global trade networks using the multimodal high-dimensional heterogeneous Transformer (MH-DP Transformer) architecture has provided significant insights into the complex dynamics of global trade. The experimental results demonstrate the effectiveness of the proposed model in capturing nonlinear cascading effects and dynamic chain reactions, outperforming other comparative models in path prediction accuracy and convergence speed. The model’s ability to integrate multimodal data and extract high-dimensional features has allowed for a more comprehensive understanding of the interactions between major economies, revealing the intricate propagation mechanisms of trade shocks and the asymmetry in global trade flows. The visualization of trade flow changes through 3D charts and heat maps further enhances the interpretability of the model, providing a valuable tool for policymakers and businesses to anticipate and respond to global trade fluctuations.

Despite the promising results of the current study, there are several directions for future research. Firstly, the model could be further optimized to enhance its adaptability and robustness, particularly in highly volatile and complex economic environments. This might involve refining the architecture to better handle extreme economic events and improve prediction performance during such periods. Secondly, incorporating more granular data sources could potentially increase the model’s accuracy and provide a finer-grained analysis of global trade networks. Additionally, exploring the integration of machine learning techniques with the existing framework could further improve the model’s ability to capture the dynamic nature of global trade. Finally, extending the model to include real-time data processing capabilities would enable more timely and responsive predictions, making it a more powerful tool for decision-makers in the fast-paced global economy.

## 6. Conclusion

This article proposes a method for analyzing the multi-path effects within global trade networks using a multimodal high-dimensional heterogeneous Transformer architecture. By leveraging innovative cascading effect modeling and dynamic chain reaction analysis, it uncovers the intricate propagation mechanisms within global trade flows. The experimental results demonstrate that the proposed method significantly improves the accuracy of trade path predictions and highlights the critical role of multi-path effects in maintaining global trade stability. However, there are certain limitations in the current approach, such as its reduced predictive performance during extreme economic events. Future research could focus on optimizing the model to enhance its adaptability, robustness, and predictive capabilities, particularly in highly volatile and complex economic environments. Additionally, exploring the integration of more granular data sources and incorporating machine learning techniques could further improve the model’s effectiveness in capturing the dynamic nature of global trade networks.
